# Chloroplasts and Plant Sustainability: Key Roles and Emerging Insights

**DOI:** 10.3390/ijms27114675

**Published:** 2026-05-22

**Authors:** Nunzia Scotti, Rachele Tamburino

**Affiliations:** 1 CNR-IBBR, National Research Council of Italy, Institute of Biosciences and BioResources, Via Università 133, 80055 Portici, NA, Italy; 2 CNR-IBE, National Research Council of Italy, Institute of Bioeconomy, Via P. Gobetti 101, 40129 Bologna, Italy; rachele.tamburino@cnr.it

**Keywords:** plastids, crops, climate change, resilience, defence

## Abstract

Chloroplasts are the primary sites of photosynthesis, but growing evidence highlights their broader role as central hubs that coordinate plant responses to environmental challenges. They retain a semi-autonomous genetic system and communicate extensively with the nucleus through anterograde and retrograde signalling pathways, enabling coordinated cellular regulation. Beyond energy conversion, chloroplasts host key biosynthetic pathways and dynamically adjust their metabolic and redox states in response to developmental and environmental cues. This review summarizes the current knowledge of chloroplast functions in response to abiotic and biotic stresses, emphasizing their contribution to plant resilience, productivity and sustainability. Under abiotic stress, chloroplasts undergo structural, metabolic and redox reprogramming to maintain photosynthetic efficiency and metabolic homeostasis. During biotic stress, they act as a powerful signalling platform that integrates immune responses with metabolic and redox regulation. These functions rely on overlapping signalling pathways that are differentially tuned to support acclimation or defence. By coordinating stress responses with photosynthetic activity and metabolic efficiency, chloroplasts play a central role in sustaining plant productivity and represent promising targets for enhancing crop resilience and agricultural sustainability under climate change and increasing pathogen pressure.

## 1. Introduction

Plastids originated from a photosynthetic bacterium closely related to modern cyanobacteria that was engulfed by a eukaryotic cell. This primary endosymbiotic event not only explains the origin of photosynthetic plastids but also established the basis for the later functional diversification of plastid types. They are surrounded by a double membrane and contain a small genome (i.e., plastid genome or plastome) that encodes for up to 120–130 genes required for plastid function. During evolution, most genes from the ancestral endosymbiotic bacterium were transferred to the nucleus, giving rise to a semi-autonomous organelle that depends on the synthesis of thousands of nuclear-encoded proteins in the cytosol and their subsequent import into the organelle for proper functionality [1,2]. Because many protein complexes involved in plastid functions contain subunits encoded by nuclear and plastid genomes (e.g., Rubisco, photosystems I and II), their expression is finely regulated through two different mechanisms known as anterograde and retrograde signalling. Anterograde regulation involves communication from the nucleus to the plastid via nuclear-encoded proteins that control plastid differentiation and homeostasis. Conversely, plastids generate retrograde signals according to their developmental and functional state, thereby modulating nuclear gene expression and various cellular processes [3,4,5,6].

Plastids share many features with cyanobacteria, including sequence homology, translational machinery, and the fatty acid biosynthesis pathway [7,8,9]. Although the chloroplast is the best-known plastid type, a wide range of biosynthetic pathways, such as those involved in the synthesis of amino acids, lipids, hormones, vitamins, antifungal toxins, and bactericidal compounds, occur across different plastid forms [4].

Among plastids, chloroplasts are unique in their ability to harvest light energy and convert it into energy-rich organic compounds. They contain the most abundant soluble and membrane proteins in plants (i.e., Rubisco and LHCII, respectively) as well as the most abundant photosynthetic pigments and lipids, including chlorophylls and monogalactosyldiacylglycerol, respectively [10]. In addition, chloroplasts can also dynamically adjust their energy-converting and metabolic performance in response to the metabolic demands of the plant, as well as developmental and environmental cues [11,12]. Because they integrate energy conversion, metabolism and stress signalling, chloroplasts function as the primary site of stress perception that regulates responses to developmental and environmental signals, thereby acting as key determinants of plant resilience and sustainability. Consequently, extensive research has focused on elucidating the role of chloroplasts in mediating stress adaptation and integrating signals during pathogen infection and plant defence responses, with the aim of understanding how plants maintain growth, defence and productivity under adverse conditions [11].

This review summarizes current knowledge on the chloroplast as an environmental sensor that integrates photosynthesis, stress signalling and metabolic reprogramming and highlights how chloroplast functions contribute to plant resilience, productivity and sustainability under fluctuating environmental conditions.

## 2. Photosynthesis

Photosynthesis is a complex and remarkably inefficient process that sustains life on Earth by converting solar energy and atmospheric CO_2_ into organic compounds through the coordinated action of numerous genes and biosynthetic pathways. Due to its high sensitivity to environmental and biological perturbations, the chloroplast acts as an early stress sensor, translating external stimuli into redox and metabolic signals that trigger adaptive and defensive molecular responses. These responses strongly influence photosynthetic efficiency and plant resilience, ultimately imposing major limitations on crop productivity [10,13,14,15]. Therefore, improving photosynthetic performance represents a promising strategy for enhancing crop productivity and plant sustainability under climate change conditions.

**Figure 1:**
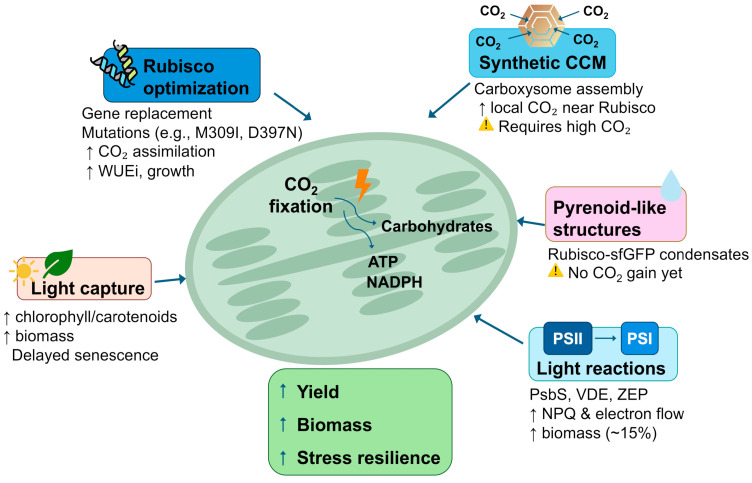
A schematic representation of main strategies pursued to improve photosynthesis and carbon assimilation (see details in the main text). This figure was created using Microsoft PowerPoint per Mac (version 16.109.1). Icons were downloaded from the free website https://www.magnific.com (accessed on 26 March 2026).

In recent years, numerous studies have focused on different aspects of the photosynthetic process, including the accumulation of photosynthetic pigments, photochemical quenching, light energy conversion, carbon fixation (Figure 1). These studies collectively demonstrate that improving photosynthesis remains a promising strategy for enhancing crop productivity, sustainability and resilience [10,16,17].

Ribulose-1,5-bisphosphate carboxylase oxygenase (Rubisco), the most abundant protein on Earth, plays a crucial role in carbon fixation but is an extremely inefficient enzyme. For this reason, it has long been a primary target of genetic engineering aimed at improving carbon fixation, particularly in C3 plants, which lack the carbon-concentrating mechanism found in C4 species [18]. To better understand the determinants of Rubisco kinetics, gene replacement approaches have been widely used [19,20,21,22,23,24]. Replacement of the tobacco *rbcL* gene by plastid transformation with plant- [20,21,22,23,24] or archaeal-derived genes [19] generally produced transplastomic plants with defective phenotypes (e.g., pale-green or slower growth), decreased enzyme activity, or a requirement for elevated CO_2_ levels. In contrast, specific point mutations (M309I and D397N) in the *rbcL* gene, introduced using chloroplast base editors (ptpTALECD or ptpTALECD_v2mod) in *Arabidopsis thaliana*, led to improvements in several photosynthetic parameters, such as CO_2_ assimilation rate, electron transport rate (PSII), intrinsic water-use efficiency (WUEi) and overall plant growth under both ambient and elevated CO_2_ concentrations [25].

More recently, Chen et al. [26,27] explored alternative strategies to enhance carbon fixation based on the engineering of carboxysomes or inducing the condensation of endogenous Rubisco in tobacco chloroplasts. Many organisms (e.g., autotrophic bacteria, algae, C4 plants) have CO_2_-concentrating mechanisms (CCMs) around Rubisco. In cyanobacteria and many proteobacteria, these mechanisms rely on carboxysomes, protein-based structures composed of hexameric, pentameric and trimeric protein assemblies that encapsulate Rubisco with a fast turnover rate and carbonic anhydrases (CA). Within carboxysomes, bicarbonate (HCO_3_^−^) is actively transported into the cell, accumulates in the cytosol, and is converted by CA into CO_2_, thereby increasing CO_2_ concentration at Rubisco catalytic sites and favouring carboxylation. In their study, Chen et al. [27] introduced a complete set of α-carboxysome components into the tobacco plastome, encoded by nine genes from the cso operon of the proteobacterium *Halothiobacillus neapolitanus*. The resulting chloroplast-expressed carboxysomes displayed structural and functional properties comparable to native counterparts. Transplastomic plants were capable of autotrophic growth and completed their life cycle under air supplemented with 1% CO_2_ (*v*/*v*), although growth was slower than in wild-type plants and not sustainable under ambient CO_2_ conditions. These findings indicate that further optimization is required, including improved carboxysome assembly and structure, the expression of active bicarbonate transporters, and elimination of endogenous chloroplastic CA to enable efficient HCO_3_^−^ accumulation.

A second strategy to improve photosynthesis and carbon assimilation was based on the condensation of endogenous Rubisco within tobacco chloroplasts by fusing the superfolder green fluorescent protein (sfGFP) to the tobacco Rubisco large subunit (RbcL). Due to the intrinsic oligomerization properties of sfGFP, pyrenoid-like Rubisco condensates with dynamic, liquid-like behaviour were generated. Importantly, the C-terminus fusion did not impair Rubisco holoenzyme assembly or activity. Unlike carboxysome-expressing transplastomic plants, these chloroplast-engineered plants exhibited normal autotrophic growth and completed their life cycle under ambient conditions, with no significant phenotypic differences compared to wild-type plants. However, no increase in net CO_2_ assimilation rates was observed between transplastomic and wild-type plants, indicating that further investigation is necessary to improve Rubisco-sfGFP catalytic efficiency to translate this strategy into enhanced crop productivity [26].

Several studies have investigated the impact of increased accumulation of photosynthetic pigments on photosynthesis and agronomic traits [26,27,28,29,30,31,32]. For example, overexpression of transcription factors regulating chlorophyll biosynthesis (CCT39, a member of the CONSTANS, CONSTANS-LIKE and TIMING OF CAB EXPRESSION 1 (CCT) family) or repressing chloropyll degradation (OBP2a, a member of the OBF-BINDING PROTEIN family) increased chlorophyll content, photosynthetic capacity and plant biomass in *Populus* sp. [26,27], and delayed senescence in radish [32], respectively. Similarly, Wang et al. [31] showed that overexpression of a newly identified dual regulator from *Capsicum annuum* (CaBBX10) in tomato, associated with the biosynthesis of both chlorophylls and carotenoids, enhanced chlorophyll levels in mature-green fruits and increased total carotenoid content, including lycopene, β-carotene and violaxantin in red-ripe fruits [31].

Improvements in the light reactions have also been achieved through the simultaneous overexpression in tobacco of three photoprotective proteins from *A. thaliana*: the photosystem II integral membrane protein (PsbS), the violaxanthin de-epoxidase (VDE), which converts violaxanthin into zeaxanthin, and the zeaxanthin epoxidase (ZEP), which catalyzes the reverse reaction. These plants displayed an improved photoprotective response to natural shading, resulting in increased leaf carbon dioxide uptake and plant dry matter productivity by about 15% in fluctuating light [30]. Although PsbS plays a key role in regulating the transition of the light-harvesting complex II (LHCII) into the photoprotective, energy-dissipative state (qE), its precise molecular mechanism has remained unclear. Recent mutagenesis studies identified critical amino acid residues (E67 and E173) involved in PsbS activation, revealing that photoprotection is associated with dynamic changes in its oligomeric state and conformation [26].

Similarly, overexpression of the tobacco Rieske protein (PETC), a component of the cytochrome *b_6_f* complex, enhanced PSI and PSII quantum efficiency, electron transport rates, biomass accumulation, and seed yield in transgenic *Arabidopsis thaliana* [33].

Overall, these findings indicate that, despite substantial progress, additional integrative approaches are still needed to successfully convert improvements in photosynthetic processes into stable increases in crop productivity under field conditions.

## 3. Abiotic Stress

**Figure 2:**
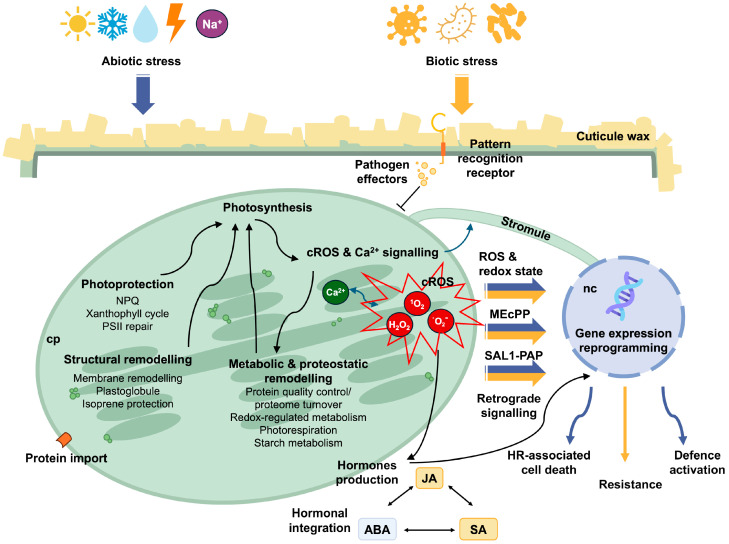
Chloroplast as a central hub integrating major abiotic and biotic stress responses. Under abiotic stress, perturbation of photosynthesis triggers thylakoid membrane remodelling, photoprotective responses, metabolic reprogramming and redox signalling, promoting acclimation. During biotic stress, chloroplasts amplify immune signalling through cROS (chloroplast-derived) production, Ca^2+^ dynamics, defence hormones biosynthesis and stromule-mediated connections with the nucleus. Chloroplast-derived retrograde signals (including ROS, redox carriers, PAP (3′−phosphoadenosine 5′−phosphate) and MEcPP (2−C−Methyl−D−erythritol–2,4–cyclophosphate)), coordinate nuclear gene expression. The frequent targeting of chloroplast functions by pathogen effectors highlights their role as key nodes in plant-pathogen interactions. Blue and yellow arrows indicate abiotic and biotic stress-related pathways, respectively. nc: nucleus; cp: chloroplast; NPQ: Non Photochemical Quencing; PSII: photosystem II; ROS: Reactive Oxygen Species; Ca^2+^: calcium ions; ABA: abscissic acid; JA: jasmonic acid; SA: salicylic acid; HR–associated cell death: hypersensitive response-associated cell death. This figure was created using Microsoft PowerPoint per Mac (version 16.109.1). Icons were downloaded from the free website https://www.magnific.com (accessed on 26 March 2026).

Environmental perturbations rapidly impact photosynthetic processes and metabolic homeostasis [34,35,36,37,38]. In response, chloroplasts initiate coordinated structural, metabolic, redox, and signalling adjustments that promote stress acclimation and modulate nuclear gene expression through plastid-to-nucleus (retrograde) signalling pathways, as summarized in Figure 2. These responses are essential not only for plant survival but also for maintaining photosynthetic efficiency, metabolic balance and productivity under fluctuating environmental conditions. A comparative overview of major abiotic stress types, investigated species, and the associated chloroplast structures, functions and regulatory processes is provided in Table 1.

Abiotic stresses such as drought, salinity, high light, heat and cold directly affect chloroplast structure and function, often through remodelling of thylakoid membranes. Changes in the composition and unsaturation level of thylakoid membrane lipids, including monogalactosyldiacylglycerol (MGDG), digalactosyldiacylglycerol (DGDG), sulfoquinovosyldiacylglycerol (SQDG) and phosphatidylglycerol (PG), together with increased fatty acid desaturation mediated by chloroplast-localized desaturases, enhance the proportion of polyunsaturated galactolipids and are crucial to maintaining membrane fluidity, photosystem stability and efficient electron transport under stress conditions [36,39,40,41,42,43].

Consistent with this structural plasticity, plastoglobules, monolayer lipid droplets associated with thylakoid membranes, undergo dynamic changes in size, number and protein composition in response to salinity, high light, heat and drought, contributing to lipid remodelling, redox regulation and photoprotection across multiple species [44]. Regulated adjustments in thylakoid membrane fluidity further enhance tolerance to light and heat stress in Arabidopsis, reinforcing the concept that chloroplast structural organization is actively controlled during acclimation [45]. Additional evidence of membrane adaptation under stress involves volatile isoprenoids. As reviewed by Zuo et al. [46], isoprene and related compounds synthesized via the chloroplast methylerythritol phosphate (MEP) pathway help maintain membrane integrity under heat stress, thus contributing to thermotolerance. In Arabidopsis and *Platanus orientalis* isoprene emission stabilizes thylakoid ultrastructure, particularly stacked grana regions, following heat exposure. Moreover, isoprene emission is associated with reduced accumulation of chloroplast-derived Reactive Oxygen Species (cROS) and sustained photosynthetic efficiency in several species, including *Nicotiana tabacum* and *Vismia guianensis*. Conversely, suppression of the isoprene synthase gene (ISPS) in *Populus × canescens* leads to increased ROS accumulation and reduced photosynthetic electron transport and CO_2_ assimilation under heat stress [46].

Similarly, salinity stress affects chloroplast ultrastructure, although responses are species-dependent. In glycophytic plants such as *Arabidopsis thaliana*, rice, wheat and spinach, salt stress is commonly associated with chloroplast swelling, partial unstacking of grana, dilation of the thylakoid lumen, increased plastoglobule number and size, and, in severe cases, disruption of envelope integrity and reduced starch accumulation. In contrast, halophytic species such as *Thellungiella* and *Atriplex* generally maintain thylakoid organization and exhibit controlled plastoglobule remodelling, reflecting adaptive structural plasticity that helps sustain photosynthetic performance under high salinity [47].

Beyond structural changes, chloroplasts rapidly activate photoprotective mechanisms to cope with excess energy and oxidative pressure. Dynamic regulation of non-photochemical quenching (NPQ), carotenoid composition and the xanthophyll cycle dissipates excess excitation energy, modulates photosynthetic electron flow and protect sphotosystems under high light and heat stress in Arabidopsis and major crops such as wheat, rice, maize and barley [36,48]. In addition to their role in energy dissipation, xanthophylls such as zeaxanthin act as redox regulators, preventing over-reduction in the photosynthetic electron transport chain and limiting ROS formation.

At the signalling level, abiotic stress perturbs photosynthetic electron transport, leading to increased production of cROS, including singlet oxygen (^1^O_2_), superoxide (O_2_^−^) and hydrogen peroxide (H_2_O_2_), as demonstrated in Arabidopsis and rice under high light, drought and salt stress [49,50,51]. ROS production within chloroplasts is both spatially and chemically specific. Singlet oxygen is mainly produced at photosystem II, whereas superoxide and hydrogen peroxide originate primarily from photosystem I and stromal redox reactions [52]. Although excessive ROS accumulation causes photoinhibition, lipid peroxidation, protein oxidation and loss of photosynthetic capacity, controlled ROS production acts as a key signalling mechanism that activates antioxidant defences and promotes acclimation through transcriptional and metabolic reprogramming [52]. In particular, ROS-dependent redox dynamics directly modulate thiol-based redox systems, including thioredoxin and peroxiredoxin networks, which fine-tune the activity of several stromal enzymes and link redox status to carbon fixation and metabolic flux. Indeed, reversible cysteine oxidation regulates key Calvin-Benson cycle enzymes (e.g., fructose–1,6–bisphosphatase, sedoheptulose–1,7–bisphosphatase, glyceraldehyde–3–phosphate, etc.), as well as ATP synthase activity and photosystem repair processes [52]. In addition to thiol-based regulatory systems, chloroplast redox homeostasis is maintained by enzymatic antioxidant networks, among which the ascorbate-glutathione cycle represents a major pathway for ROS detoxification in both the stroma and thylakoid membranes [52]. This cycle involves the coordinated action of superoxide dismutase, ascorbate peroxidase, monodehydroascorbate reductase, dehydroascorbate reductase and glutathione reductase, enabling efficient scavenging of hydrogen peroxide and regeneration of reduced antioxidants in the chloroplast stroma and thylakoid membranes [49,53].

Chloroplast-derived ROS also play a central role in plastid-to-nucleus communication (retrograde signalling). In particular, chloroplast-derived H_2_O_2_, can diffuse or be transmitted to the nucleus via defined signalling routes, coordinating nuclear stress-responsive gene expression with chloroplast metabolic status [52,54]. Similarly, singlet oxygen involves the chloroplast-localized EXECUTER1 (EX1) and EXECUTER2 (EX2) proteins, whose oxidative modification and FtsH-dependent turnover trigger the induction of singlet oxygen-responsive nuclear genes. Changes in the redox state of the plastoquinone (PQ) pool reflect the balance between light harvesting and electron consumption and influence the expression of photosynthesis-related nuclear genes. In addition to ROS, plastid-derived metabolites contribute to retrograde signalling during abiotic stress. Accumulation of 3′-phosphoadenosine 5′-phosphate (PAP) in the SAL1-PAP pathway links chloroplast redox imbalance to nuclear transcriptional reprogramming, thereby enhancing tolerance to drought, high light and heat stress [55,56,57]. Similarly, the singlet oxygen-derived apocarotenoid β-cyclocitral (β-CC), generated from β-carotene oxidation at PSII, modulates oxidative stress responses, while the MEP pathway intermediate 2–C-Methyl–D–erythritol–2,4–cyclophosphate (MEcPP), integrates plastid metabolic status with nucleus transcriptional responses [58]. Tetrapyrrole intermediates, such as Mg–protoporphyrin IX and heme, have also been proposed to contribute to plastid–to–nucleus signalling under stress conditions [59,60].

In parallel with ROS signalling, abiotic stress induces rapid changes in chloroplast–associated calcium (Ca^2+^) dynamics. Heat, high light, and salinity trigger transient increases in chloroplasts Ca^2+^ levels, including thylakoid-linked Ca^2+^ fluxes, consistent with the role of thylakoid membranes in Ca^2+^ buffering and signalling [35,61]. The thylakoid-localized Calcium-Sensing receptor (CAS) acts as a key component in chloroplast Ca^2+^ signalling, linking Ca^2+^ dynamics to photosynthetic electron transport and downstream transcriptional responses [62]. In the stroma, Ca^2+^ signals are decoded by Ca^2+^-binding proteins such as calmodulin and calmodulin-like proteins, which interact with redox-sensitive enzymes to fine-tune photosynthetic activity and stress acclimation [35,52,61].

Photorespiration represents another important chloroplast-associated acclimation mechanism under abiotic stress, functioning as a metabolic safety valve that dissipates excess reducing power when CO_2_ fixation is limited, as occurs during drought, heat or salinity [63]. Chloroplast carbohydrate metabolism also contributes to stress acclimation. Environmental perturbations frequently alter the balance between carbon fixation and carbohydrate utilization, leading to adjustments in starch biosynthesis and degradation. Starch turnover acts as a dynamic buffer for excess photosynthate and helps maintain metabolic and redox balance. In addition, chloroplast-derived sugars and sugar-phosphate intermediates participate in cellular signalling networks that coordinate energy status, growth and stress responses [64,65].

Further, chloroplasts contribute to hormonal regulation during abiotic stress, as several stress-related hormones or their precursors are synthesized within this organelle. Under drought, salinity and high-temperature, abscisic acid (ABA) biosynthesis is strongly induced, promoting stomatal closure, activation of antioxidant defences, and transcriptional reprogramming that enhances stress tolerance [36]. This highlights the role of chloroplasts in the coordination of metabolic and hormonal responses required for acclimation under adverse environmental conditions.

Abiotic stress also modulates chloroplast gene expression and RNA metabolism. Both plastid-encoded genes and nucleus-encoded transcription factors that regulate plastid transcription, RNA processing and translation, exhibit stress-specific regulation under drought, high light, heat and cold conditions [12,66]. Chloroplast gene expression is largely controlled at post-transcriptional level, including RNA editing, splicing, stabilization, turnover, and translational regulation. These processes are mediated by nuclear-encoded chloroplast-localized RNA-binding proteins, such as pentatricopeptide repeat (PPR) proteins, chloroplast ribosome maturation (CRM) proteins, DEAD-box RNA helicases and S1-domain containing proteins [40,66,67,68,69,70,71].

Beyond rapid redox regulation, cROS drive long-term proteome reorganization by activating protein quality control pathways, including ATP-dependent proteases and molecular chaperones that preserve protein integrity under stress [52]. Furthermore, chloroplast proteome composition is dynamically adjusted through regulation of nuclear-encoded protein import via the TOC-TIC translocon system, whose activity is modulated according to organellar folding and repair capacity [72]. Under stress conditions this process involves selective control of import efficiency, including ubiquitin- and autophagy-mediated turnover of TOC components, as shown in *A. thaliana* under UV-B irradiation and heat stress [72,73,74]. Consistently, proteomic and transcriptomic analyses in tomato and potato showed that drought stress alters the abundance of photosynthetic proteins, redox enzymes, chaperones and protein quality control components, changes closely linked to ABA accumulation, redox homeostasis and genotype-dependent recovery capacity [75,76].

Taken together, these processes enable chloroplasts to maintain photosynthetic efficiency, metabolic balance and resource use efficiency, thus sustaining plant growth under adverse environmental conditions.

**Table 1:** Chloroplast processes involved in plant responses to abiotic stress ^1^.

Abiotic Stress	Species Studied	Chloroplast Function/Structure Involved	Main Chloroplast Processes/Signals	Type of Signal	Reference
Salt stress	Arabidopsis, rice, halophytes	Chloroplast ultrastructure	Thylakoid remodelling, plastoglobules	Lipid remodelling	[47]
Salt, high light, heat, drought	Arabidopsis; rice; maize; tomato	Plastoglobules	Lipid metabolism, photoprotection	Lipid remodelling/redox	[44]
Cold stress	Arabidopsis	Thylakoid membranes	Fatty acid desaturation	Lipid remodelling	[42]
Temperature stress	Arabidopsis	Thylakoid membrane lipids	Fatty acid unsaturation	Lipid remodelling	[43]
Heat stress	*Populus* sp., *Nicotiana tabacum*, *Quercus* spp.	Isoprenoid biosynthesis (MEP pathway)	Isoprene/monoterpenes, membrane stabilization, ROS reduction	Lipid/membrane stabilization	[46]
Heat/light stress	Arabidopsis	Thylakoid membrane fluidity	PSII protection and repair	Membrane stability	[45]
High light/fluctuating light	*Arabidopsis thaliana*	Thylakoids	NPQ, PSII repair, ROS generation and signalling	ROS/photoprotection	[51]
High light	*Arabidopsis thaliana*	Pigment metabolism	Xanthophyll cycle, carotenoids,	Photoprotection	[36,48]
Cold stress	*Arabidopsis thaliana*	Thylakoid quality control	FtsH protease, PSII repair, ^1^O_2_ signalling	ROS signalling/proteostasis	[77]
Multiple stresses	Arabidopsis	Chloroplast redox network	ROS-mediated signalling and proteostasis	ROS signalling	[52]
Multiple stresses	Arabidopsis	cROS network	ROS-mediated retrograde signalling and proteostasis	ROS signalling	[54]
General abiotic stress	Crops	Antioxidant systems	Ascorbate-glutathione cycle	Antioxidant/ROS detox	[53]
Salt stress	Arabidopsis	cROS network	H_2_O_2_ retrograde signalling	ROS signalling/retrograde	[78]
Combined stresses		ROS and NO signalling	Redox signalling networks	ROS/NO signalling	[79]
High light, oxidative stress	*Arabidopsis thaliana*	Retrograde signalling	ROS, β-cyclocitral, MEcPP, PAP	Retrograde signalling	[80]
Drought, high light	*Arabidopsis thaliana*	Retrograde signalling	SAL1–PAP pathway, ROS/ABA coordination		[55,56]
Drought stress		Chloroplast signalling network	ROS, Ca^2+^ oscillations, PAP and MEcPP retrograde signal	ROS/retrograde signalling	[81]
Multiple stresses	Arabidopsis	Chloroplast Ca^2+^ network	CAS-mediated Ca^2+^ signalling	Ca^2+^ signalling	[62]
Multiple stresses	Arabidopsis	Chloroplast Ca^2+^ dynamics	Ca^2+^ oscillations	Ca^2+^ signalling	[35]
Multiple stresses	Arabidopsis	Thylakoid Ca^2+^ buffering	Ca^2+^-dependent signalling	Ca^2+^ signalling	[61]
Multiple stresses		Starch metabolism	Starch turnover	Metabolism	[64]
Drought/metabolic stress		Chloroplast metabolism	Photorespiration	Metabolic acclimation	[63]
Multiple stresses	*Arabidopsis thaliana*, *Nicotiana tabacum*	Metabolic/redox crosstalk	Aconitase-mediated retrograde signalling	Metabolic/redox signalling	[82]
Multiple stresses	*Arabidopsis thaliana*, crops	Chloroplast proteostasis	Protein import (TOC–TIC), turnover, quality control	Proteostasis	[72,83]
Heat/UV-B stress	Arabidopsis	TOC complex	Autophagy-mediated regulation of chloroplast protein import	Proteostasis/ protein import	[73]
Drought stress	*Solanum lycopersicum*	Chloroplast proteome	Proteome remodelling, redox enzymes, ABA-linked signalling	Proteostasis	[76]
Drought stress	Rice, maize, wheat	Chloroplast-associated metabolic responses	Photosynthesis and photorespiration	Metabolic acclimation	[40]
Drought stress	*Arabidopsis thaliana*, vegetable crops (e.g., bean, sugar beet)	Chloroplast-associated metabolic responses	Photosynthesis inhibition, redox imbalance, ABA integration	Metabolic signalling	[37]
Heat stress	*Arabidopsis thaliana*, rice, wheat, maize, tomato	Chloroplast metabolism	Metabolic reprogramming, ROS production, acclimation	ROS/metabolism	[50]

^1^ Due to the heterogeneity of the experimental systems and the reported parameters, the table provides a qualitative comparative overview rather than a quantitative synthesis.

## 4. Biotic Stress

Plants rely on a multilayered innate immune system to counteract pathogen invasion, mainly based on pattern-triggered immunity (PTI) and effector-triggered immunity (ETI). PTI is activated upon the recognition of pathogen-associated molecular patterns (PAMPs) or damage-associated molecular patterns (DAMPs) by plasma membrane-localized pattern recognition receptors (PRRs), leading to the initiation of broad defence responses [84,85]. ETI, represents a second, more specialized layer of defence and is mediated by intracellular immune receptors that detect specific pathogen effector proteins, often resulting in a rapid and localized hypersensitive response (HR) that restricts pathogen proliferation [86]. PTI and ETI operate in a coordinated and synergistic manner, sharing core signalling modules and reinforcing each other to enhance defence capacity. Their activation triggers a wide range of downstream responses that partially overlap with those described for abiotic stress, including ROS production, calcium signalling and transcriptional reprogramming, but are specifically directed toward defence activation and pathogen restriction [87,88,89].

Within this framework, chloroplasts emerge as central integrative platforms that amplify defence-associated signals while maintaining cellular functionality under pathogen pressure (Figure 2). A comparative overview of pathogen types, species studied and the corresponding chloroplast structures, functions and regulatory processes involved in plant responses to biotic stress is provided in Table 2. Although several chloroplast-centred redox and retrograde signalling pathways described for abiotic stress are conserved components of immunity, pathogen infection redirects these pathways toward defence activation, including hypersensitive response (HR), systemic signalling and disease resistance [90,91,92,93].

At the structural and metabolic level, chloroplasts contribute to early defence by supplying de novo synthesized C16–C18 fatty acids that serve as precursors for the biosynthesis of long-chain fatty acids involved in cuticular wax formation, a primary physical barrier that restricts pathogen entry and influence pathogen accessibility [92,94]. In addition, chloroplast metabolism provides carbon skeletons and energy required for the synthesis of cell wall components, and other defence-associated metabolites, linking photosynthetic activity to the reinforcement of apoplastic barriers during the early stages of infection [92].

A key feature of chloroplast involvement in plant immune responses is their rapid structural repositioning and morphological remodelling. During PTI and ETI, chloroplasts accumulate around the nucleus and form stromules, highly dynamic stroma-filled tubular extensions that enhance chloroplast–to–nucleus communication by promoting the targeted delivery of ROS and defence-related proteins [95,96]. Stromule formation is supported by cytoskeleton-associated factors such as the kinesin KIS1, underscoring the functional importance of plastid dynamics during defence [96].

At the signalling level, the chloroplast functions as a major source of secondary messengers during immune responses. Chloroplast-derived ROS and chloroplast-associated Ca^2+^ fluxes, previously described in abiotic responses, are similarly integrated into immune networks, influencing kinase cascades, transcription factor activity and hormone biosynthesis [92,97,98,99,100,101]. As in abiotic stress, ROS production is spatially and functionally controlled, allowing these molecules to act as signals rather than merely cytotoxic by-products [102]. The specificity and amplitude of these signals depend strongly on redox poise and antioxidant capacity, which fine-tune chloroplast redox homeostasis and influence downstream immune outputs and hormone balance [103,104,105]. Tight regulation of chloroplast-derived immune signals is therefore essential to activate effective defence without imposing excessive metabolic costs.

During ETI, chloroplast function undergoes extensive reprogramming, including modulation of photosystem activity, restriction of carbon assimilation and alteration of plastid gene expression [106,107]. These changes promote stromal over-reduction and enhance ROS production, contributing to a redox-dependent amplification of defence responses and triggering hypersensitive cell death [106], while remaining tightly controlled by the antioxidant system and calcium signalling networks to prevent excessive oxidative damage [108]. Experimental manipulation of chloroplast redox buffering in tobacco using plastid-targeted flavodoxin demonstrated that light and chloroplast redox status strongly influence the outcome of both non-host and virulent bacterial interactions by modulating cROS levels and defence-associated metabolic and transcriptional reprogramming. Notably, this modulation affects the execution of hypersensitive cell death without broadly impairing defence gene activation [109,110]. Similarly, crop studies support a role for chloroplast-localized regulators in modulating pathogen-induced ROS dynamics and HR intensity. In wheat, the chloroplast-localized ribosome-binding GTPase TaTypA positively regulates resistance to stripe rust in association with ROS accumulation and HR [111].

As in abiotic stress responses, plastid–to–nucleus communication remains central during biotic stress, where previously described retrograde signalling pathways are re–purposed to regulate defence-related gene expression. Rather than introducing distinct signalling pathways, pathogen infection redirects chloroplast-derived signals toward the activation of immune responses and hormonal pathways. In this context, retrograde signalling contributes to the coordination of salicylic acid (SA)- and jasmonic acid (JA)-dependent pathways and the accumulation of defence-related metabolites, thus linking chloroplast functional status with immune outputs [104,105,112]. Photorespiration is also integrated into this network, contributing to H_2_O_2_ signalling and inter-organellar redox control, particularly in relation to SA- and JA-dependent responses [113,114].

The chloroplast also represents a central node for the biosynthesis and integration of defence hormones. While ABA plays a main role in abiotic stress responses, chloroplast-derived pathways also contribute to the production of other hormones that are more prominently involved in biotic stress. It is the primary site for salicylic acid precursor synthesis via the isochorismate pathway and provides lipid precursors for jasmonate biosynthesis [48,115]. Through redox-dependent regulation and chloroplast–to–nucleus communication, the chloroplast influences the balance and crosstalk between SA-, JA- and ABA-mediated pathways, thus determining defence prioritization under different pathogen pressures [92,115,116,117]. Fine control of SA signalling is further mediated by chloroplast-localized regulatory proteins; for example, post-translation modification of SA-binding proteins modulates both SA perception and feedback regulation of immune responses, and chloroplast-associated modulation of SA biosynthesis and signalling directly affects resistance to biotrophic pathogens [93]. In tomato, miRNA-dependent regulation of ROS-scavenging machinery includes effects on chloroplast Cu/Zn-superoxide dismutase and JA-related defence gene expression during *Botrytis cinerea* infection, further highlighting the tight link between cROS homeostasis and immune competence [118].

Beyond redox regulation and hormone biosynthesis, pathogen infection also affects chloroplast gene expression, translation capacity and proteostasis. Immune-induced chloroplast remodelling includes regulation of plastid translation and elongation factors (e.g., StTuA/StTuB target of *Phytophtora infestans* effector), which reshape plastid proteome composition in response to immune cues [119]. In rice, pathogen-triggered small-RNA regulatory modules can influence plastid integrity; *Rhizoctonia solani* infection has been associated with miRNA-mediated regulation of a pentatricopeptide repeat target proposed to contribute to chloroplast degradation and susceptibility [120]. Virus-chloroplast interactions provide additional mechanistic insight into how biotic stress perturbs plastid homeostasis. In *Nicotiana benthamiana* infected with *South African cassava mosaic virus*, the repression of *nitric oxide-associated 1* gene (*NOA1*) and multiple chloroplast translation factors coincides with reduced chlorophyll and carotenoid content, as well as chloroplast dysfunction underlying chlorotic symptoms [121]. In sugar beet, early infection by Beet yellows virus is associated with transcriptional deregulation that includes downregulation of chloroplast-related genes, suggesting an early impairment of chloroplast function that may contribute to symptom development [122].

Given their central role, chloroplasts are major targets of pathogen effectors that suppress chloroplast-mediated immunity. Viral and microbial proteins frequently localize to chloroplasts to interfere with photosynthetic electron transport, inhibit ROS production, alter chloroplast translational capacity and disrupt hormone biosynthesis, thereby promoting infection [90,123,124,125]. Recent work showed that nucleus-encoded chloroplast elongation factors can promote cROS production and chloroplast–nucleus communication, while also serving as direct targets of pathogen effectors, reinforcing the concept of the chloroplast as both a strategic platform for immune signal integration and a point of vulnerability in plant immunity [119]. Additional studies have further highlighted the importance of plastid translational capacity and retrograde signalling in immune regulation [91,125]. In the wheat–stripe rust pathosystem, the RING-type E3 ligase TaPIR1 promotes susceptibility by targeting TaHRP1 and suppressing chloroplast function and the expression of Photosynthesis-Associated Nuclear Genes (PhANGs), supporting the idea that pathogen success can involve active attenuation of chloroplast performance and associated immune competence [126]. Likewise, in citrus leaves infected by *Xanthomonas citri pv. citri*, a pathogen-encoded plant natriuretic peptide-like factor modulates host chloroplast and photosynthetic protein profiles, counteracting photosynthesis shutdown and prolonging tissue viability [127]. This frequent targeting of chloroplast functions underscores that the organelle represents a key battleground in plant–pathogen interactions [128], and that maintaining chloroplast integrity is critical not only for immunity but also for sustaining photosynthetic tissue viability and overall plant performance during infection.

Overall, these findings indicate that chloroplasts reprogram shared signalling and metabolic modules to prioritize defence during biotic stress. This functional flexibility allows plants to balance immune responses with the maintenance of metabolic efficiency and productivity under pathogen infection.

**Table 2:** Chloroplast processes involved in plant responses to biotic stress.

Biotic Stress/Interaction	Species Studied	Chloroplast Function/Structure Involved	Main Chloroplast Processes/Signals	Type of Signal/ Response	Reference
General immune responses (bacterial, fungal, viral)	Arabidopsis, *Nicotiana* *benthamiana*, crops	Chloroplast as immune signalling hub	Integration of PTI/ETI outputs; coordination of redox, hormonal and retrograde signals	Immune integration/retrograde signalling	[102]
Early immune activation after PAMP perception	Arabidopsis	Photosynthetic apparatus/chloroplast-associated signalling	PTI-associated perturbation of photosynthesis; immune-linked chloroplast signalling	PTI/photosynthesis crosstalk	[98,123]
PTI and ETI	Arabidopsis	Chloroplast Ca^2+^ network (CAS)	Stromal Ca^2+^ signalling; activation of SA biosynthesis genes; transcriptional reprogramming of defence genes	Ca^2+^ signalling/immune regulation	[99]
ETI/avirulent bacterial interactions	Arabidopsis	Chloroplast ROS (cROS) network	cROS accumulation linked to HR-associated programmed cell death	Redox signalling/HR	[97,100]
PTI and ETI	*Nicotiana* *benthamiana*, Arabidopsis	Stromules and chloroplast repositioning	Stromule induction; perinuclear chloroplast clustering; enhanced chloroplast-nucleus communication	Organelle dynamics/retrograde signalling	[95,96]
Pathogen infection/retrograde signalling	Arabidopsis	SAL1–PAP pathway	Regulation of glucosinolate accumulation; modulation of SA- and JA-dependent pathways; contribution to immune competence	Metabolite retrograde signalling/hormone regulation	[104]
Biotrophic/hemibiotrophic defence	Arabidopsis and model plants	SA biosynthesis in chloroplasts	Isochorismate pathway; chloroplast-derived SA precursor synthesis; immune hormone integration	Hormonal signalling (SA)	[115]
Necrotrophic defence/wound-related immunity	Arabidopsis and crops	Chloroplast fatty acid metabolism	Lipid precursors for JA biosynthesis; oxylipin-mediated defence responses	Hormonal signalling (JA)/lipid signalling	[116]
Broad immune modulation during infection	Arabidopsis, crops	Hormone integration in chloroplasts	Coordination of SA, JA and ABA crosstalk during pathogen challenge	Hormone crosstalk/signalling integration	[92,103,117]
Photorespiration during immunity	Arabidopsis, multiple pathosystems	Photorespiration	H_2_O_2_ production; inter-organellar redox control; interaction with SA/JA signalling	Metabolic signalling/redox regulation	[113,114]
Non-host and virulent bacterial interactions	Tobacco	Chloroplast redox buffering	Flavodoxin-dependent modulation of cROS; defence-associated metabolic and transcriptional reprogramming; selective control of HR	Redox signalling/defence cost modulation	[109,110]
Stripe rust resistance	Wheat	Chloroplast-localized regulator	TaTypA-dependent cROS accumulation and HR intensity	Redox signalling/HR	[111]
Viral infection/chloroplast-to-nucleus signalling	*Nicotiana* *benthamiana*	Chloroplast retrograde signalling/PhANG regulation	KPILP-mediated repression of LHCB, HEMA1, RBCS1A; altered carbon partitioning	Retrograde signalling/transcriptional regulation	[91]
Viral infection	*Nicotiana* *benthamiana*	Chloroplast translation machinery	Repression of NOA1 and plastid translation factors; chloroplast dysfunction and pigment loss	Translation/plastid homeostasis	[121]
Viral infection	Sugar beet	Chloroplast-related gene expression	Downregulation of chloroplast-associated genes during early infection	Transcriptional regulation/plastid dysfunction	[122]
Oomycete infection	Potato	Plastid translation/elongation factors	Regulation of plastid translation; cROS production; stromule-associated immune competence; effector targeting	Translation/proteostasis/effector targeting	[119]
Fungal infection	Rice	Plastid integrity/post-transcriptional regulation	miRNA-mediated regulation of PPR targets linked to chloroplast degradation and susceptibility	RNA regulation/plastid homeostasis	[120]
Bacterial infection	Arabidopsis	NECGs ^1^/PSII	Effector-mediated suppression of photosynthesis; inhibition of cROS burst and CO_2_ assimilation	Effector targeting/photosynthesis-defence trade-off	[123]
Viral infection	*Nicotiana* *benthamiana*	Chloroplast-localized viral protein	NSvc4-mediated inhibition of cROS and chloroplast-mediated defence	Effector targeting/ROS suppression	[124]
Stripe rust susceptibility	Wheat	PhANG ^2^ regulation/ chloroplast function	TaPIR1-mediated suppression of PhANGs; reduced cROS; increased susceptibility	Effector-associated suppression/transcriptional control	[126]
Citrus canker	Citrus	Photosynthetic proteins/ chloroplast proteome	Pathogen-mediated modulation of photosynthesis; maintenance of host tissue viability	Pathogen manipulation/metabolic reprogramming	[127]
General pathogen pressure	Arabidopsis, *Nicotiana* spp., crops	Chloroplast as effector target	Direct targeting of chloroplast proteins and signalling pathways by pathogen effectors	Effector targeting/immune suppression	[119,123]

^1^ NECG: nuclear-encoded chloroplast-targeted gene, ^2^ PhANG: photosynthetic-associated nuclear gene.

## 5. Conclusions

Chloroplasts are a central hub that regulates plant responses to environmental and biotic stresses by integrating metabolic and signalling pathways. Indeed, they coordinate structural, redox, hormonal and transcriptional responses, enabling plants to adapt to diverse challenges. Particularly, chloroplasts rely on a shared set of core regulatory modules (i.e., redox signalling, calcium dynamics, metabolite-mediated communication and plastid–to–nucleus retrograde signalling) that are conserved across stress conditions but are functionally reprogrammed depending on the nature of the stimulus. Under abiotic stress, these regulatory modules primarily promote acclimation by maintaining photosynthetic efficiency and metabolic homeostasis. In contrast, in response to pathogen cues, they are redirected toward defence activation, the amplification of immune signalling, and, when required, hypersensitive cell death.

Chloroplasts occupy a strategic position at the interface between energy conversion, stress signalling and growth regulation. By integrating environmental cues with metabolic and hormonal networks, they directly influence plant resilience, resource use efficiency and yield stability. Consequently, enhancing chloroplast performance represents a key strategy for developing crops capable of sustaining productivity under increasingly variable environmental conditions.

Future research should focus on elucidating how chloroplast signalling networks are coordinated under combined abiotic and biotic stresses, on the exploitation of the genetic variability, and how these processes can be engineered to enhance both stress tolerance and photosynthetic efficiency. Such advances will be essential for translating fundamental knowledge into sustainable agricultural strategies that address the challenges of climate change and rising global food demand.

## Data Availability

No new data were created or analyzed in this study. Data sharing is not applicable to this article.
